# Multicellular group formation in *Saccharomyces cerevisiae*

**DOI:** 10.1098/rspb.2019.1098

**Published:** 2019-09-04

**Authors:** R. M. Fisher, B. Regenberg

**Affiliations:** Section for Ecology and Evolution, Department of Biology, University of Copenhagen, Copenhagen, Denmark

**Keywords:** yeast, multicellularity, adhesion, major evolutionary transition

## Abstract

Understanding how and why cells cooperate to form multicellular organisms is a central aim of evolutionary biology. Multicellular groups can form through clonal development (where daughter cells stick to mother cells after division) or by aggregation (where cells aggregate to form groups). These different ways of forming groups directly affect relatedness between individual cells, which in turn can influence the degree of cooperation and conflict within the multicellular group. It is hard to study the evolution of multicellularity by focusing only on obligately multicellular organisms, like complex animals and plants, because the factors that favour multicellular cooperation cannot be disentangled, as cells cannot survive and reproduce independently. We support the use of *Saccharomyces cerevisiae* as an ideal model for studying the very first stages of the evolution of multicellularity. This is because it can form multicellular groups both clonally and through aggregation and uses a family of proteins called ‘flocculins’ that determine the way in which groups form, making it particularly amenable to laboratory experiments. We briefly review current knowledge about multicellularity in *S. cerevisiae* and then propose a framework for making predictions about the evolution of multicellular phenotypes in yeast based on social evolution theory. We finish by explaining how *S. cerevisiae* is a particularly useful experimental model for the analysis of open questions concerning multicellularity.

## 1. Introduction

Multicellular organisms dominate the world we see around us, and yet they are formed from millions of individual cells that specialize on different tasks and cooperate to form a cohesive body. Understanding how and why cells cooperate to form multicellular structures is a central aim of evolutionary biology, because multicellularity has arisen many times across the tree of life [1] and has led to the most important species radiations for both biological complexity and diversity.

**Figure 1.**
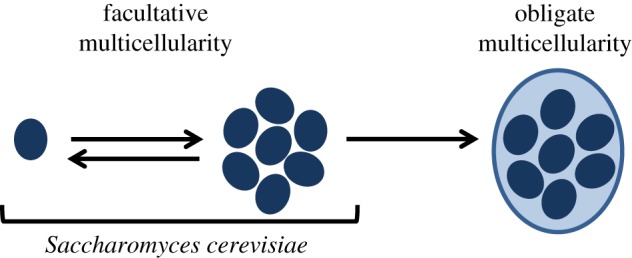
Stages of major evolutionary transitions. Major evolutionary transitions involve independent units (genes, cells, or organisms) joining together to form a social group, which then becomes a new individual through the evolution of mutual dependence [2]. *Saccharomyces cerevisiae* is able to form multicellular groups through cooperation, but remains facultatively multicellular—i.e. it has not made the major evolutionary transition to obligate multicellularity [3,4]. (Online version in colour.)

**Figure 2.**
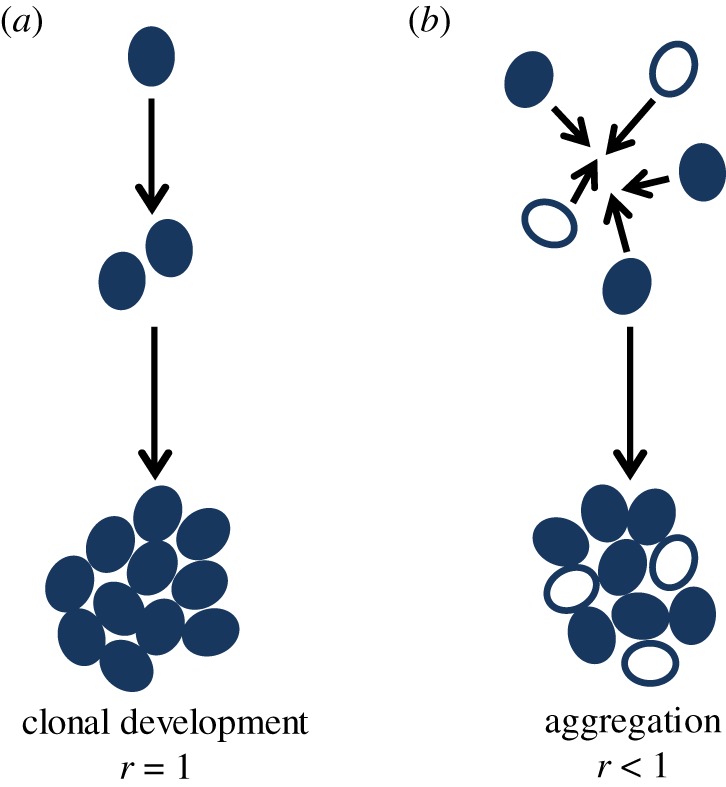
Modes of multicellular group formation. (*a*) Cells can form multicellular groups through clonal development, where daughter cells remain attached to mother cells after cell division. This guarantees that cells will be clonally related to each other (relatedness, *r* = 1). (*b*) Cells can aggregate to form a multicellular group of cells. These can be genetically similar or dissimilar cells (relatedness, *r* < 1). (Online version in colour.)

The evolution of obligate multicellularity, like we see in animals and plants, has been called a ‘major evolutionary transition in individuality’ because cells are entirely mutually dependent on each other and conflict between them is so minimal that they can be considered a new individual (figure 1) [5,6]. However, this transition has only ever occurred in species that have clonal multicellular development [7], meaning cells will be genetically identical, leading to clonal relatedness, which can happen when daughter cells remain attached to mother cells after division (figure 2*a*). Multicellularity can also arise as a consequence of cell aggregation, however this has never led to obligate multicellularity (figure 2*b*). For example, the slime mould *Dictyostelium discoideum* and other species that form groups through aggregation remain able to switch between unicellularity and multicellularity, making them facultatively multicellular (figure 1). As a consequence, they have a lower number of cell types and are generally smaller, compared to species that form multicellular groups through clonal development [7].

There is a growing and convincing pool of evidence suggesting that the way in which multicellular groups form is key for understanding when and how major evolutionary transitions occur, through its effect on relatedness between the interacting cells (figure 2) [6–9]. However, it is hard to study major evolutionary transitions by focusing only on obligately multicellular organisms, because the factors that favour multicellular cooperation cannot be disentangled, as cells cannot survive and reproduce independently (figure 1). Obligately multicellular species may have also undergone secondary changes that make the origins of multicellularity unclear. Hence, factors that favour multicellularity are best studied in facultative multicellular species. Many examples of this are found across the tree of life, but very few concrete examples exist where species are able to form multicellular groups through both aggregation and clonal development, making it difficult to investigate the mechanisms and consequences of the two types of group formation experimentally in one species.

Here, we propose bakers yeast, *Saccharomyces cerevisiae*, as an ideal model for studying the very first stages of the evolution of multicellularity as a major evolutionary transition in individuality (figure 1). This is because: (i) it is able to switch between unicellularity and multicellularity, (ii) it can do this through both modes of group formation (clonal development and aggregation), and (iii) it is a well-studied, genetically tractable model organism. In this paper, we briefly review current knowledge about group formation and multicellularity in *S. cerevisiae* and propose a framework for making predictions about the evolution of multicellular phenotypes in yeast based on social evolution theory. We suggest terminology that is general and useful, and we finish by suggesting outstanding questions and potentially fruitful avenues for future research.

## 2. Why is group formation important?

The way in which multicellular groups form has fundamental consequences for behaviour, complexity, and social evolution, because it has direct implications on the genetic relatedness between interacting cells [6,7]. When groups form through aggregation, cells are likely to be genetically different and so the resulting multicellular group will contain cells that are genetically unrelated (or at least non-clonal) (figure 2*b*). In contrast, when groups form through cell division, by the daughter cell remaining attached to the mother, cells will be clonally related to each other (figure 2*a*). Relatedness is known to be an important force shaping social behaviour, as cells that are genetically related will be more likely to engage in cooperative behaviours, compared to cells that are unrelated [10]. For example, *Pseudomonas aeruginosa* show higher levels of cooperative siderophore production when they are interacting with relatives, compared to when they are interacting with non-relatives [11].

One pervasive problem with the evolution of cooperation is the potential of genetically different cheats to invade groups of cooperators and reap the benefits of cooperation without paying costs. Cheating has been recognized as a major challenge to explaining the evolution of cooperative behaviours among cells [12]. The exclusion of cheats is a major hurdle that groups of cells must overcome in order to maintain cooperation and ensure the benefits of cooperation are returned to other cooperative cells [12,13]. Hence, the way in which the multicellular groups form will have a profound influence on whether or not cheats even have the potential to invade.

There is also compelling comparative evidence that clonal relatedness (resulting from clonal group formation) between cells has always been a necessary condition for the evolution of complex, obligate multicellularity like we see in animals and plants, and some lineages of fungi and algae [7].

## 3. Multicellularity in yeast: a major evolutionary transition?

**Figure 3.**
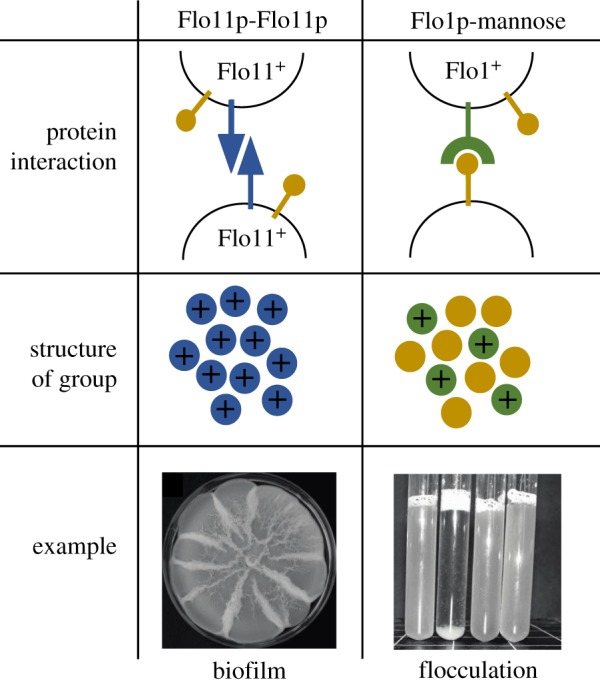
Flocculins determine the structure of multicellular groups. Homophilic (self-self) and heterophilic (self-non-self) interactions of Flo11p and other flocculins (Flo1p, Flo5p, Flo9p, and Flo10p). The left panel shows the way Flo11p (coloured in blue) adheres to other Flo11p on neighbouring cell walls. Flo11p does not interact directly with mannose residues (coloured in yellow). Flo11p will only adhere to Flo11p, creating self-self adherence and therefore clonal groups of cells. An example of this can be found in clonal biofilms. The right panel shows the way Flo1p (coloured in green) can adhere to mannose residues which are expressed by all cells, meaning multicellular groups can contain cells of different genotypes. Images: surface spreading biofilm on semi-solid complex growth medium after two weeks growth (as described in strain CLIB326 [15]) and flocculation of diploid yeast in liquid complex medium (second tube from left, strain from left to right: *SFL1*/*sfl1^Q320STOP^*; *sfl1^Q320STOP^*/*sfl1^Q320STOP^*; *sfl1^Q320STOP^*/*SFL1*; *SFL1*/*SFL1* in the CEN.PK strain background described in [16]). (Online version in colour.)

How do yeasts fit into this framework? Yeast are a polyphyletic group of species within the Kingdom Fungi. They are predominantly unicellular, although many yeasts are known to switch between unicellular and multicellular lifestyles depending on environmental factors, so we classify them as facultatively multicellular (see Glossary). Yeasts have evolved at least five times independently within the Kingdom Fungi [14] and many of the most important fungal pathogens and biotechnologically useful species are yeasts. *S. cerevisiae* is perhaps the most famous, displaying a startling variety of natural multicellular phenotypes, including pseudohyphae, biofilms, and flocs (figure 3) [3,17–19] that are also common in other yeasts [20]. In the laboratory, they generally grow as single cells often because researchers have selected for unicellular phenotypes that are easier to work with [21].

Despite adaptations to a multicellular lifestyle, including many genes involved in adhesion, *S. cerevisiae* remains facultatively multicellular [3]. This means that many natural isolates of *S. cerevisiae* can readily form multicellular groups from single cells in response to external stimuli (e.g. nutrient limitation [17,19,22] or high alcohol concentrations [23]). This is always flexible and never a permanent transition to multicellularity, where individual cells are obligately part of a multicellular body and can never survive and reproduce outside the multicellular body. Because of this, and despite its astonishing diversity of multicellular behaviour, *S. cerevisiae* should not be considered as having undergone a major evolutionary transition to multicellularity (figure 1, Glossary).

## 4. How does *Saccharomyces cerevisiae* become multicellular?

In order to be multicellular, cells need to be able to adhere to one another. In natural isolates of *S. cerevisiae*, adhesion is conferred by a family of proteins called flocculins. They comprise seven different functional *FLO* genes, coding for five proteins involved in multicellularity [24,25] and two proteins specific for conjunction of haploid cells in mating [26]. Flocculins are cell wall proteins that are anchored to the cell membrane and protrude from the cell wall to confer cell–cell and cell–surface adhesion [27].

In *S. cerevisiae*, flocculins can be broadly split into two types, based on the structure of their amino terminal A-domain (the part of the protein responsible for adhesion [3]). Flo1p, Flo5p, Flo9p, and Flo10p confer general adhesion, by sticking to mannose residues that protrude from the surfaces of other cells [28,29]. Although Flo1p cells have higher affinity to other Flo1p cells, they also adhere to cells not producing flocculins [23]. In contrast, Flo11p confers very specific adhesion, through a homophilic Flo11p-Flo11p interaction and is expressed during growth [30] (figure 3). So, while the other flocculins make cells generally ‘sticky’, *FLO11* produces a protein that will only adhere to other cells expressing *FLO11* [15]*.* Furthermore, the daughter receives *FLO11* mRNA from her mother during development and because *FLO11* is expressed during growth, adhesion between mother and daughter cells is ensured [31].

Therefore, flocculins in *S. cerevisiae* produce two distinct ways of sticking together and forming multicellular groups (figure 2). Flo1p, Flo5p, Flo9p, and Flo10p result in aggregative multicellular group formation — cells expressing them will stick to other cells in a general ‘sticky’ response regardless of their genotype. On the other hand, expression of Flo11p will lead to clonal group formation between related cells, usually a mother and daughter cell after division. This special quality of the flocculins found in *S. cerevisiae* means that flocculin expression corresponds almost exactly to two distinct ways of forming multicellular groups (figure 2); aggregative and clonal group formation.

### (a) Flocs

Flocculation was initially described for *S. cerevisiae* in wine and beer making, where yeast cells form aggregates when sugar levels drop that are often visible to the naked eye (figure 3) [32]. Flocculation potentially protects the yeast cells from harsh environmental conditions — strains of *S. cerevisiae* that flocculate show increased resistance to ethanol and oxidative stress [23].

Flocs are therefore a particularly useful industrial trait in the brewing process, allowing yeast to be removed from cultures easily at low glucose concentrations and high ethanol concentrations. Several different flocculin genes are expressed during flocculation, including *FLO1*, *5*, and *10* (table 1) and they are produced by cells adhering to other cells in the environment, rather than through cell division.

**Table 1.** The genetic basis of multicellularity in yeast. Flocculin genes involved in multicellular group formation in *Saccharomyces cerevisiae*, adhesive properties of the flocculins, the multicellular phenotypes produced, and the way in which multicellular groups are formed.

gene	adhesive properties	multicellular phenotype	mechanism of group formation	references
*FLO1*	heterophilic cell–cell adhesion through mannose residues	flocculation	aggregation (non-clonal)	[24,33,34]
*FLO5*	heterophilic cell–cell adhesion through mannose residues	flocculation	aggregation (non-clonal)	[33,35]
*FLO9*	heterophilic cell–cell adhesion through mannose residues	flocculation	aggregation (non-clonal)	[27]
*FLO10*	heterophilic cell–cell adhesion through mannose residues	flocculation	aggregation (non-clonal)	[24,27]
*FLO11*	homophilic cell–cell adhesion through Flo11p on other cells and cell–surface adhesion	biofilms, pseudohyphae, flocculation	cell division (clonal)	[19,22,24,36,37]

### (b) Biofilms

Biofilm is a broad term for multicellular structures that form on surfaces either in a liquid environment or surface-spreading biofilms in a liquid–air interphase. Biofilms are seen in many species of both bacteria and yeasts, and can be comprised of a single species or multiple species [38]. *Saccharomyces cerevisiae* forms both surface spreading biofilms and biofilms in liquid environments that are dependent on Flo11p [19,39] ensuring mother–daughter cell adhesion. *Saccharomyces cerevisiae* biofilms aid in colonization of new environments, for the monopolization of nutrients and adhesion to surfaces [15,19]. It is also possible they can protect against anti-fungals through the presence of slow- and non-growing cells [40,41].

Surface-spreading biofilms on semi-solid 0.3% agar are particularly interesting because of the large variety of growth forms found in natural isolates [15,42]. Recently, Regenberg *et al.* [15] showed that when grown on semi-solid 0.3% agar, certain strains of *S. cerevisiae* form differentiated biofilms. Such biofilms are created through a *FLO11* epigenetic switch where both Flo11^+^ and Flo11^−^ cells are produced simultaneously in one population of cells [3,15,43]. These biofilms outgrow others without the epigenetic Flo11 switching mechanism, and appear not to mix with other biofilm colonies, thereby maintaining clonality when they encounter other colonies. This research shows that conditional differentiation between adhesive and non-adhesive cells can allow cells to outgrow competitors through cooperation in a multicellular biofilm and that differentiation between cells might be selected for in very early stages of multicellularity, but without necessarily leading to obligate multicellularity.

### (c) Pseudohyphae

Pseudohyphal growth is a filamentous growth form that allows diploid cells of *S. cerevisiae* to grow on a surface in nitrogen-limited environment with little increase in their biomass [17]. Pseudohyphae are comprised of a chain of elongated cells that remain attached after unipolar budding. As with biofilm formation, the Flo11p protein is essential for pseudohyphal growth [36] (table 1), presumably ensuring adhesion between mother and daughter cells and thereby producing clonality in the pseudohyphae colony.

Unlike many other species, *S. cerevisiae* is able to form multicellular groups both by aggregation and through cell division, resulting in different multicellular phenotypes (figure 3). When groups are formed through budding, as is the case for biofilms and pseudohyphae, the cells in the multicellular group will be clonal (all else being equal). However, flocculation can occur between genetically dissimilar cells, meaning that relatedness will be less than clonal and variable. Therefore, the way in which these various multicellular groups form has consequences for cell–cell relatedness, and this means we can make several predictions about the social interactions we may expect.

## 5. Multicellularity and susceptibility to cheating

Biofilms and pseudohyphae, where cells are clonally related to one another, should intrinsically be able to withstand the effects of cheating, simply because the way in which the groups form will exclude cheating cells (as shown [15]). This is because Flo11^+^ cells can only adhere to other cells expressing Flo11^+^ (figure 3) and Flo11^+^ daughter cells do not separate from their Flo11^+^ mother cells. This means clonal biofilms expressing *FLO11* have the inherent capacity to protect against invasion by other genotypes. This is not the case for flocculation. This is because flocs are formed through aggregation of potentially unrelated cells (figure 2*b*) [23]. Cells in the floc adhere to each other through expression of *FLO1*, but the flocculating Flo1^+^ cells can be of different origin, leading to flocs comprised of genetically different cells. Non-producers can still adhere because Flo1p is able to stick to mannose residues produced by all cells, not just by other producers (figure 3) [23]. Cheats could therefore reap the benefits of flocculating without paying the cost of expressing *FLO1*. In fact, there is evidence that loss-of-function mutants can occur in and spread through natural populations of yeast expressing the *FLO1* homologue, *FLO5* [44]. This provides support for the observation that aggregation doesn't lead to multicellular organismality because multicellular groups forming through aggregation can pick up disruptive selfish mutants during aggregation [2].

## 6. Facultative multicellularity in *Saccharomyces cerevisiae*

One of the characteristics that makes *S. cerevisiae* so useful for studying the first stages of multicellularity is that it is facultatively multicellular, allowing us to examine both the factors favouring multicellular group formation and the genetic and molecular mechanisms involved. *Saccharomyces cerevisiae* uses multicellularity to adapt quickly to changing environments, using a combination of genetic and epigenetic mechanisms to produce adaptive variability in its adhesive properties, and it predominantly does this through effects on genes for flocculin proteins.

Transcription of *FLO11* is regulated by yeast pheromones, pH, glucose, amino acids, and nitrogen sources [16,45–49] (extensively reviewed by Brückner & Mösch, 2011 [3]). Besides their regulation by sugars [50], *FLO1*, *FLO5*, *FLO9*, and *FLO10* are carried in sub-telomeric regions of the chromosomes [27,51] that are normally low in transcription, preventing expression of the flocculin genes [24]. The fact that *S. cerevisiae* regulates the *FLO g*enes in this way suggests that there has been selection for variability in multicellular phenotypes. In other words, it seems likely that there has been a selective pressure for *S. cerevisiae* to be unicellular under some conditions and multicellular under other conditions and therefore be able to switch between unicellularity and multicellularity through expression of flocculins.

In *S. cerevisiae*, genes encoding flocculins have intragenic repeats that encode the middle domain of the flocculins [3]. Comparison of the *FLO11* middle domain between strains of *S. cerevisiae* reveals that the number of repeats and length of the middle domain varies substantially between *FLO11* orthologues, revealing a large variation between Flo11p and suggesting fast evolution of *FLO11* [52]. This means that flocculin genes can change size quickly and frequently, providing the genetic basis for substantial functional diversity in the adhesive properties of *Saccharomyces* strains [52]. The common explanation for expansion and contraction of short repeats is replication slippage, but meiotic or mitotic recombination between slightly displaced *FLO* alleles could also explain both expansions and contractions [53]. Moreover, a screen for extrachromosomal circular elements in *S. cerevisiae* revealed the existence of circular DNA elements consisting of the repeats of *FLO11* as well as *FLO1* [54], which might be intermediates in repeat deletions and/or expansions. The expansion and contraction of *FLO* genes in *S. cerevisiae* is therefore likely to allow quick adaptive adjustment to changing environmental conditions [52].

More unusually, it seems as though prions may play a role in modulating the flexibility of multicellular phenotypes in *S. cerevisiae* [55]. Many natural strains carry prions, and are able to switch between prion and non-prion states. In addition, *FLO11* regulators have a high propensity to produce prions [55], meaning that prion switching through its effects on the *FLO11* gene, allows flexibility in the production of multicellular phenotypes [55].

## 7. *Saccharomyces cerevisiae* in the laboratory

*Saccharomyces cerevisiae* has been used historically as a model for eukaryotic genetics due to the ease by which it can be cultured in the laboratory and by which it lent itself to genetic studies of gene linkage [56,57]. A major advantage of using *S. cerevisiae* as a model is the valuable strain collections and genetic tools developed by a large community of yeast geneticists over the past 30 years, which makes *S. cerevisiae* one of the most well understood eukaryotic organisms at the molecular level [39,58–61].

There are several aspects in particular that make *S. cerevisiae* a desirable and tractable model organism for studying the evolution of multicellularity. Firstly, there are robust methods for the transformation of *S. cerevisiae* with exogenous DNA, and making all types of chromosomal mutations including insertions, deletions, and substitutions [56]. Furthermore, there are now mutant strain collections where any one of the approximately 6000 genes have been deleted in otherwise functional strains [39,58]. One strain collection is made in the Σ1278b genetic background that expresses *FLO11* naturally, which has allowed the identification of genes and proteins involved in biofilm and pseudohyphal growth [22,39].

Secondly, most yeast proteins can be tagged with fluorescent markers (GFP, RFP, etc.) so that phenotypes of interest can be visualized through fluorescent microscopy [59]. This allows researchers to see the cellular level structure of multicellular phenotypes such as biofilms, and to investigate how cells expressing different adhesive properties interact. Finally, the sequenced genomes of *S. cerevisiae* strains allow comparative genomics studies [61,62] that have already revealed that, for example, *FLO1* and *FLO11* are among the fastest-evolving genes in the yeast genome [52].

These methods, among others, mean that we can ask important questions about the evolution of multicellularity using *S. cerevisiae* that may not be possible with other organisms. For example, phenotypes and behaviours found in nature can be manipulated and studied in genetic tractable strains [39].

## 8. Experimental evolution of multicellularity

Recently, *S. cerevisiae* has been used as a model for studying the molecular and environmental factors that can select for multicellularity [13,15]. Experimental evolution and genetic engineering using laboratory populations of *S. cerevisiae* has resulted in the evolution of multicellularity [63–65] through settling selection (centrifugation and gravity, [63]) and continuous cultures [65]. Murray and co-workers have furthermore shown that multicellularity provides a selective advantage for exploitation of public goods [64,66]. The researchers exploited *S. cerevisiae*'s ability to grow on sucrose through secretion of the sucrose-degrading enzyme, invertase, and found that low concentrations of sucrose select for the evolution of multicellularity [66]. The molecular basis of multicellularity in these experiments rely on mutations allowing expression of *FLO* genes [65] or loss-of-function mutations in genes involved in mother–daughter cell separation (e.g. *ACE2*, [65,67], *AMN1*, and a chitinase gene, [66]). More advanced experiments with mutants that are differentiated into two cell types, show that multicellularity can stabilize differentiation, making groups of differentiated cells less susceptible to cheating [13]. Although these are artificial systems, the speed with which simple multicellularity evolved, with the disruption of just one or a few genes, shows the genetic capacity and flexibility of *S. cerevisiae* to respond to its external environment using multicellularity.

## 9. Concluding remarks

There has been a wealth of research on multicellularity in yeast on mechanisms [28–30] and social evolution [4,15,23,63,64]. However, we suggest that there is an opportunity to synthesize this research within the major evolutionary transitions framework and to capitalize on an incredibly useful experimental system for studying the first stages of multicellularity.

We believe that multicellular group formation in *S. cerevisiae*, through the expression of flocculins, provides an ideal system for studying multicellularity. Firstly, the facultative nature of multicellularity in *S. cerevisiae* means it is possible to study and manipulate the benefits and costs of group formation in controlled experiments. Secondly, flocculin proteins allow us to study the effect of different modes of group formation on multicellular cooperation. For example, we can use the flocculin proteins that confer aggregative (e.g. Flo1p) and clonal (Flo11p) adhesion as an opportunity to study the effect of different modes of group formation on cooperative behaviours in the same species. This could provide a complementary laboratory system to the comparative research showing how crucial group formation is in determining subsequent multicellular evolution.

## Data accessibility

This article has no additional data.

## Glossary

Multicellularity: When multiple individual cells are in contact. This includes cells sticking together transiently through production of a sticky substance, coordinated groups of cells that show cooperative behaviours such as the production of public goods, and obligate groups of cells forming multicellular organisms like we see in animals and plants.
Obligate multicellularity: When individual cells are obligately part of a multicellular body, and cannot survive and reproduce outside of the multicellular body. Obligate multicellularity is developmentally determined, and not a response to environmental conditions.
Facultative multicellularity: When individual cells can become part of a multicellular body in response to environmental conditions, and then can revert to being unicellular again. They do not rely on being multicellular in order to survive and reproduce. Facultative multicellularity include both simple forms, where cells stick to each other to form a clump (e.g. some bacterial biofilms) to more complex forms with differentiated cells (e.g. slime moulds and ciliates).
Major evolutionary transition in individuality: A major evolutionary transition in individuality occurs when individual units (e.g. genes, cells, or individuals) cooperate and form a new, more complex individual, that can subsequently only reproduce as a whole.
Multicellular organism: An obligately multicellular species that has undergone a major evolutionary transition in individuality.
